# Enterohemorrhagic *Escherichia coli* Excretion by Child and Her Cat

**DOI:** 10.3201/eid1302.061106

**Published:** 2007-02

**Authors:** Ulrich Busch, Stefan Hörmansdorfer, Stephan Schranner, Ingrid Huber, Karl-Heinz Bogner, Andreas Sing

**Affiliations:** * Bavarian Health and Food Safety Authority, Oberschleißheim, Germany; † Veterinary Inspection Office, Landshut, Germany

**Keywords:** Enterohemorrhagic *Escherichia coli*, EHEC, cat, infection cycle, letter

**To the Editor:** Enterohemorrhagic *Escherichia coli* (EHEC) can cause severe hemorrhagic colitis characterized by gastrointestinal symptoms and bloody diarrhea as well as hemolytic uremic syndrome (*1*). Cattle and small ruminants are the major natural reservoir of these foodborne pathogens (*1*,*2*). Human infections may also develop after direct contact with cows, goats, sheep, and deer (*1*). Although domestic dogs and cats are known as rare EHEC carriers (*3*,*4*), no human EHEC infections associated with pet contact have been reported. Here we report the first case of an EHEC strain infecting both a child and her domestic cat.

A 2-year-old girl with bloody diarrhea and vomiting subsequently tested positive for EHEC serotype O145:H–. The isolated strain harbored the pathogenicity-associated genes *stx1*, *stx2*, *eae,* and *hly,* as tested by PCR. An enterohemolytic phenotype was also present. After notification of the local health authority, a rigorous search for the possible source of the girl’s infection was started. When asked for instances of animal contact, her parents mentioned the family cat, which the girl often handled. The cat is restricted to the house, has no contact with other animals, and is fed only canned catfood. The animal strictly uses a litter box, which is cleaned regularly by the parents. No gastrointestinal symptoms in the cat were recorded. Repeated stool samples from the cat grew a strain of EHEC O145:H– that showed the identical pathogenicity gene pattern as the girl’s isolate. Moreover, a restriction fragment length polymorphism analysis proved the clonal identity of both strains. Because both the girl and the cat continuously excreted the EHEC strain, the cat was assumed to be a possible source of the girl’s infection or reinfection. The cat’s’ infection was treated with probiotics, but the child’s EHEC positivity did not change. After 3 months, the girl spontaneously stopped excreting EHEC, while the cat’s stool samples remained EHEC positive. The cat was then treated by peroral autovaccination with the heat-inactivated EHEC strain for 10 consecutive days and subsequently stopped shedding EHEC. In the Table, the clinical course and laboratory findings of both girl and cat are summarized.

**Table:** Clinical picture and isolation of EHEC serotype O145:H– from stool samples of child and her cat*

Date	Girl	Cat
Dec 1, 2004	Vomiting and diarrhea	
Dec 9	Tested positive	ND
Dec 22	Tested positive	Tested positive
Dec 28	Tested positive	ND
Jan 10, 2005	Tested positive	ND
Jan 17	Tested positive	ND
Jan 21	Tested negative	ND
Jan 24	Tested positive	Tested positive, treated with probiotics
Feb 1	Tested positive	ND
Mar 4	Tested negative	ND
Mar 12	Tested negative	ND
Apr 25	ND	Tested positive
Jun 25–Jul 4	ND	Autovaccination
Jul 29	ND	Tested negative
Aug 11	ND	Tested negative

*EHEC, enterohemorrhagic *Escherichia coli*; positive and negative refer to the isolation of EHEC serotype O145:H– ; ND, no testing was done.

To our knowledge, this case is the first documented of an EHEC strain’s affecting both a human and a domestic cat. Both excreted EHEC for ≈3 months. Although the girl had vomiting and diarrhea, the cat was asymptomatic. Several possibilities regarding the infectious process can be noted. First, the girl might have contracted the disease from her asymptomatic pet. Although in a study on *eae*-positive *E. coli* strains, ≈6% of the investigated 62 cats tested positive, none of these cats was infected with EHEC serotype O145:H– (*3*); this finding indicates that in our case the cat might not have been the direct source for the girl’s infection. Moreover, foodborne transmission to the cat seems unlikely because it was exclusively fed with canned food that was heated during preparation. Second, the cat might have been infected by the girl. Although the prevalence of EHEC serotype O145:H– is relatively low, it ranks among the 6 most often isolated non–O157 EHEC strains in human infections, accounting for 5%–7% of all non–O157 EHEC strains in prevalence studies in Finland (*5*), Germany (*6*), and the United States (*2*,*7*). A similar epidemiologic pattern for EHEC serotype O145:H– is seen in cattle (*2*,*8*). Taken together, the prevalence of EHEC serotype O145:H– in cats, humans, and cattle might indicate that the girl was probably more likely the infection source for the cat than vice versa. Third, a cycle of mutual infection and reinfection between the girl and her pet cat cannot be ruled out. Although the excretion rate for EHEC changes over time and EHEC can therefore remain undetected in stool samples while still present within the patient, the child tested EHEC negative for a short period. Despite all the precautions taken, the girl may have been reinfected by the cat.

This case illustrates several issues: 1) domestic animals such as cats (*3*), dogs (*3*,*4*), and rabbits (*9*) may serve as reservoirs for EHEC, irrespective of whether they are the primary or secondary source for these bacteria; 2) domestic cats as carriers may excrete EHEC for a prolonged period; 3) autovaccination may be effective for treating EHEC-infected animals; and 4) fondness for pets may be problematic: although EHEC O145:H– is among the 4 most often isolated EHEC serotypes associated with severe colitis or life-threatening hemolytic uremic syndrome (*10*), the girl’s parents, after weighing the infectious risks against the psychological benefits for both their daughter and her feline companion, decided not to send the cat to an animal shelter until its EHEC infection disappeared.
